# Cold-Dependent Expression and Alternative Splicing of Arabidopsis Long Non-coding RNAs

**DOI:** 10.3389/fpls.2019.00235

**Published:** 2019-02-28

**Authors:** Cristiane P. G. Calixto, Nikoleta A. Tzioutziou, Allan B. James, Csaba Hornyik, Wenbin Guo, Runxuan Zhang, Hugh G. Nimmo, John W. S. Brown

**Affiliations:** ^1^Plant Sciences Division, School of Life Sciences, University of Dundee, Dundee, United Kingdom; ^2^Institute of Molecular, Cell and Systems Biology, College of Medical, Veterinary and Life Sciences, University of Glasgow, Glasgow, United Kingdom; ^3^Cell and Molecular Sciences, The James Hutton Institute, Dundee, United Kingdom; ^4^Information and Computational Sciences, The James Hutton Institute, Dundee, United Kingdom

**Keywords:** long non-coding RNA, primary microRNA, alternative splicing, diel time-course, high-resolution RNAseq, cold transcriptome

## Abstract

Plants re-program their gene expression when responding to changing environmental conditions. Besides differential gene expression, extensive alternative splicing (AS) of pre-mRNAs and changes in expression of long non-coding RNAs (lncRNAs) are associated with stress responses. RNA-sequencing of a diel time-series of the initial response of *Arabidopsis thaliana* rosettes to low temperature showed massive and rapid waves of both transcriptional and AS activity in protein-coding genes. We exploited the high diversity of transcript isoforms in AtRTD2 to examine regulation and post-transcriptional regulation of lncRNA gene expression in response to cold stress. We identified 135 lncRNA genes with cold-dependent differential expression (DE) and/or differential alternative splicing (DAS) of lncRNAs including natural antisense RNAs, sORF lncRNAs, and precursors of microRNAs (miRNAs) and *trans*-acting small-interfering RNAs (tasiRNAs). The high resolution (HR) of the time-series allowed the dynamics of changes in transcription and AS to be determined and identified early and adaptive transcriptional and AS changes in the cold response. Some lncRNA genes were regulated only at the level of AS and using plants grown at different temperatures and a HR time-course of the first 3 h of temperature reduction, we demonstrated that the AS of some lncRNAs is highly sensitive to small temperature changes suggesting tight regulation of expression. In particular, a splicing event in *TAS1a* which removed an intron that contained the miR173 processing and phased siRNAs generation sites was differentially alternatively spliced in response to cold. The cold-induced reduction of the spliced form of *TAS1a* and of the tasiRNAs suggests that splicing may enhance production of the siRNAs. Our results identify candidate lncRNAs that may contribute to the regulation of expression that determines the physiological processes essential for acclimation and freezing tolerance.

## Introduction

Non-coding RNAs (ncRNAs) are a diverse set of RNAs which do not generally code for proteins. They include families of house-keeping ncRNAs and their precursors such as small nuclear ribonucleoprotein particle RNAs (snRNAs), small Cajal body RNAs (scaRNAs), ribosomal RNAs (rRNAs), transfer RNAs (tRNAs), and small nucleolar RNA (snoRNAs) (Shaw and Brown, 2012; Cech and Steitz, 2014). The regulatory ncRNAs include small RNAs such as microRNAs (miRNAs), short-interfering RNAs (siRNAs), and long non-coding RNAs (lncRNAs) which are expressed from intergenic regions or introns and include natural antisense transcripts (NATs). Some lncRNAs are precursors of small RNA production: primary transcripts are processed to miRNAs or siRNAs such as trans-acting siRNAs (tasiRNAs) or natural antisense siRNAs (nat-siRNAs) derived from double-stranded RNA molecules. The regulatory ncRNAs function in a wide range of cellular processes, from the regulation of transcription and splicing, to chromatin modification, gene inactivation, and translation (Liu et al., 2015). In plants and animals, tens of thousands of lncRNAs are now routinely detected in RNA-seq analyses demonstrating a new level of complexity of gene expression (Liu et al., 2012, 2015; Zhang and Chen, 2013; Yuan et al., 2016; Lu et al., 2017; Wang et al., 2017; Severing et al., 2018; Zhao et al., 2018).

In plants, the majority of lncRNAs are transcribed by RNA polymerase II (Pol II) but some are generated with Pol IV and/or Pol V (Liu et al., 2015). Like mRNAs, they are usually capped at the 5′ end, can be spliced and they form two classes which are either polyadenylated or non-polyadenylated (Liu et al., 2012; Di et al., 2014; Liu et al., 2015). Genome-wide analyses have identified thousands of plant lncRNAs in different plant species such as Arabidopsis, rice, maize, tomato, wheat, cucumber, poplar, cassava, and cotton and they are shown to be differentially expressed in response to stresses caused by abiotic environmental factors such as cold, heat, drought, and salt (Ben Amor et al., 2009; Liu et al., 2012; Di et al., 2014; Shuai et al., 2014; Zhang et al., 2014; Hao et al., 2015; He et al., 2015; Wang et al., 2015a,b; Chen et al., 2016; Forestan et al., 2016; Khemka et al., 2016; Lu et al., 2016; Yuan et al., 2016; Zhao et al., 2016; Li et al., 2017; Shumayla et al., 2017;Wang et al., 2017; Severing et al., 2018; Zhao et al., 2018). Infection of different plant species by pathogens also leads to differential expression (DE) of lncRNAs: for example, wheat with powdery mildew and stripe rust (Xin et al., 2011; Zhang et al., 2013), Arabidopsis with *Fusarium oxysporum* (Zhu et al., 2014), *Brassica napus* with stem rot (Joshi et al., 2016), and tomato with Tomato Yellow Curly Leaf Virus (Wang et al., 2018). The function of the vast majority of lncRNAs is unknown but, in general, they are primarily associated with regulation of gene expression via a range of different mechanisms and ultimately are important in differentiation, development, and abiotic and biotic stress responses (Ben Amor et al., 2009; Liu et al., 2012, 2015; Zhang and Chen, 2013; Wang et al., 2017; Severing et al., 2018; Zhao et al., 2018). The functions of specific lncRNAs illustrate the breadth of regulation of different plant processes and their modes of action (Kim and Sung, 2012; Liu et al., 2012, 2015; Zhang and Chen, 2013; Qin et al., 2017; Wang et al., 2017; Kindgren et al., 2018; Zhao et al., 2018). For example, lncRNAs are involved in vernalization-mediated regulation of flowering (Swiezewski et al., 2009; Ietswaart et al., 2012), circadian regulation of flowering (Henriques et al., 2017), low temperature suppression of flowering (Zhao et al., 2018), lateral root development (Bardou et al., 2014), gametophyte development (Wunderlich et al., 2014), photoperiod-sensitive male sterility (Ding et al., 2012), phosphate homeostasis (Franco-Zorrilla et al., 2007), cold acclimation (Kindgren et al., 2018), and drought (Qin et al., 2017).

Low temperatures negatively affect plant growth and development and, in general, plants from temperate climatic regions can tolerate chilling temperatures (0–15°C) and increase their freezing tolerance by prior exposure to low, non-freezing temperatures. This process of acclimation involves complex physiological, biochemical, and molecular changes that protect cell integrity during exposure to freezing. The multiple, cold-responsive changes reflect reprogramming of gene expression involving chromatin modification, transcription, post-transcriptional processing, post-translational modification, and protein turnover (Thomashow, 2010; Knight and Knight, 2012; Zhu, 2016). For many years, the major focus of transcriptome reprogramming in Arabidopsis in response to cold has been at the level of transcription and identifying gene targets of specific transcription factors (e.g., C-repeat binding factors – CBFs and the CBF regulon; Thomashow, 2010; Knight and Knight, 2012; Zhu, 2016). High-throughput RNA-sequencing now enables investigation of other aspects of expression control including alternative splicing (AS) and lncRNAs. AS of protein-coding pre-mRNAs is a regulated process and a major level of post-transcriptional regulation of expression; it has been associated with many different stress responses including cold (Leviatan et al., 2013; Staiger and Brown, 2013; Calixto et al., 2016; Hartmann et al., 2016; Laloum et al., 2017; Pajoro et al., 2017). Recently, the scale and dynamics of the contribution of AS to the cold response has been demonstrated by coincident waves of transcription and AS occurring in the first few hours of exposure to cold and the speed and sensitivity of some genes to small temperature changes (Calixto et al., 2018). The importance of AS in both stress responses and development is to increase proteome diversity by generating AS isoforms which encode different functional protein variants. However, AS also can regulate expression of genes by modulating the proportion of protein-coding transcripts vs transcripts which contain premature termination codons (PTCs), many of which are targeted for degradation by non-sense-mediated decay (NMD; Kalyna et al., 2012; Drechsel et al., 2013; Fu and Ares, 2014; Lee and Rio, 2015).

Little is known about the extent of AS of plant lncRNAs *per se* or how AS can affect the levels or function of lncRNAs. Many lncRNAs contain introns which are spliced during lncRNA biogenesis (Liu et al., 2012) and a number of annotated (TAIR) lncRNAs have splice variants. The best-characterized AS of a plant lncRNA is the differential AS and alternative polyadenylation of *COOLAIR* (antisense to *FLOWERING LOCUS C*), which determine the levels of FLC (Swiezewski et al., 2009; Marquardt et al., 2014). The lncRNA, *FLORE*, is regulated by the circadian clock and promotes flowering by repressing *CYCLING DOF FACTOR 5* (*CDF5*) to allow expression of FT (Henriques et al., 2017). *FLORE* is differentially expressed and alternatively spliced in response to cold but the function of *FLORE* AS is unknown. On the other hand, lncRNAs can affect the splicing/AS of target genes by binding and sequestering specific splicing factors (Bardou et al., 2014); reviewed in Romero-Barrios et al. (2018). Primary miRNA transcripts (pri-miRNAs) are a class of lncRNAs where splicing and AS have been well characterized and shown to be important to the production of some mature miRNAs (Brown et al., 2008; Yan et al., 2012; Bielewicz et al., 2013; Barciszewska-Pacak et al., 2015; Knop et al., 2017; Stepien et al., 2017). Recently, in human, deep RNA-seq targeting very low expressed RNAs found that virtually all lncRNAs are alternatively spliced generating massive lncRNA diversity which may be important to the evolution of regulatory modules of expression (Deveson et al., 2018). The intrinsic potential of splicing/AS of lncRNAs to modify their function, and of lncRNAs to influence splicing/AS and expression of target genes through a variety of mechanisms suggests a need to increase our understanding of the interplay between lncRNAs and AS.

We recently performed an ultra-deep RNA-seq analysis of a diel time-series of Arabidopsis plants transferred to cold (Calixto et al., 2018; Supplementary Figure S1). The analysis used AtRTD2, a new Arabidopsis transcriptome with greatly increased numbers of transcripts compared to the Arabidopsis TAIR10 and Araport11 databases (Zhang et al., 2017). The greater number and diversity of transcripts allowed the identification of nearly 2500 cold-regulated differentially alternatively spliced protein-coding genes, the majority of which were novel to the cold response. The time-series showed (1) massive peaks of transcription and AS of thousands of protein-coding genes in the first few hours of exposure to cold, (2) defined early and late, transient, and adaptive changes in expression and AS, and (3) demonstrated the speed and sensitivity of AS of particular genes (Calixto et al., 2018). We have interrogated this extensive time-series dataset to examine the transcriptional and post-transcriptional regulation of expression of lncRNAs and the relative contributions and dynamics of transcriptional and AS. We identify cold-responsive lncRNAs in terms of both significant DE and AS.

## Materials and Methods

### Plant Material and Growth Conditions

Details of the plant material and growth conditions have been described previously (Calixto et al., 2018). Briefly, *Arabidopsis thaliana* Col-0 seeds were grown hydroponically for 5 weeks in Microclima environment-controlled cabinets (Snijders Scientific), maintaining 20°C, 55% relative humidity, and 12 h light:12 h dark (James et al., 2012). Arabidopsis rosettes (9–13) were harvested and pooled at each sampling time forming one biological replicate. Harvesting occurred every 3 h beginning with the last 24 h at 20°C, and on days 1 and 4 after transfer to 4°C giving 26 time-points in the time-series (Supplementary Figure S1A). Day 1 at 4°C represents the “transition” from 20 to 4°C when plants first begin to experience the temperature decrease; day 4 at 4°C represents “acclimation” where plants have been exposed to 4°C for 4 days (Supplementary Figure S1A). Three biological replicates were generated for each time-point in separate experiments (78 samples in total). The switch to 4°C from 20°C was initiated at dusk. In a temperature reduction, the cabinet used here typically takes 2 h to reach 4°C air temperature. To analyze the speed of change in expression and AS, three biological replicates each consisting of 9–13 of 5-week-old rosettes were harvested just before reducing the temperature (0 min, 20°C) and after 90 min (5°C) and 120 and 180 min (4°C). Tissue was rapidly frozen in liquid N_2_ and stored at -80°C until isolation of RNA and preparation of cDNA.

### RNA Extraction, Library Preparation, and Sequencing

Total RNA was extracted from Arabidopsis tissue using the RNeasy Plant Mini Kit (Qiagen), followed by either on-column DNase treatment (for HR RT-PCR, see below), or the TURBO DNA-free^TM^ Kit (Ambion) (for library preparation and RT-qPCR, see below). RNA-seq libraries were constructed by following instructions for a TruSeq RNA library preparation (Illumina protocol 15026495 Rev. B). In these preparations, polyA+ selection was used to enrich for mRNA, RNA was fragmented for 8 min at 94°C, and random hexamers were used for first-strand cDNA synthesis. The libraries had an average insert size of approximately 280 bp and each library was sequenced on Illumina HiSeq 2500 platform generating 100 bp paired-end reads. Residual adaptor sequences at both 5′ and 3′ ends were removed from raw reads using cutadapt version 1.4.2^1^ with quality score threshold set at 20 and minimum length of the trimmed read kept at 20. The “–paired-output” option was used to keep the two paired read files synchronized and avoid unpaired reads. The sequencing files before and after the trimming were examined using fastQC version 0.10.0.

### Quantification of Transcripts and AS

Arabidopsis transcript expression from the RNA-seq data was carried out using Salmon version 0.82 (Patro et al., 2017) in conjunction with AtRTD2-QUASI augmented by eight genes that were not originally present (Zhang et al., 2017). For indexing, we used the quasi-mapping mode to build an auxiliary k-mer hash over k-mers of length 31 (–type quasi –k 31). For quantification, the option to correct for the sequence specific bias (“–seqBias”) was used. The number of bootstraps was set to 30 and all other parameters were on default settings. Transcript expression results are provided in Calixto et al. (2018) and expression profiles in the time-series data are available at https://wyguo.shinyapps.io/atrtd2_profile_app/.

### Differential Gene Expression (DE) and AS (DAS) Analysis of the RNA-seq Data

The pre-processing of the read data and DE and differential AS analyses are described in detail in Calixto et al. (2018). Briefly, transcript and gene read counts were generated from transcripts per million (TPM) data and low expressed transcripts that did not have ≥ 1 counts per million (CPM) in three or more samples were removed. At the gene level, if any transcript passed the expression level filtering step, the gene was included as an expressed gene and then the normalization factor was estimated using the weighted trimmed mean of *M* values method using edgeR version 3.12.1 (Robinson et al., 2010). Batch effects between biological replicates were estimated using RUVSeq R package version 1.4.0 with the residual RUVr approach (Risso et al., 2014). Normalized read counts in CPM were then log2 transformed and mean-variance trends were estimated and weights of variance adjustments were generated using the voom function in limma version 3.26.9 (Law et al., 2014, 2016; Ritchie et al., 2015). General linear models to determine DE at both gene and transcript levels were established using time and batch effects of biological replicates as factors and 18 contrast groups were set up where corresponding time-points in the day 1 and day 4 at 4°C blocks were compared to those of the 20°C block (e.g., block2.T1 vs block1.T1, block2.T2 vs block1.T2; Calixto et al., 2018). Genes were significantly DE at the gene level if they had at least two contrast groups at consecutive time-points with adjusted *p* < 0.01 and greater than twofold change in expression in each contrast group. Genes with significant DAS had at least two consecutive contrast groups with adjusted *p* < 0.01 and with these contrast groups having at least one transcript with ≥10% change in expression.

### Identification of lncRNA Genes

For accurate DE and differential AS analyses of lncRNAs, only those genes in AtRTD2 were selected. AtRTD2 is a new Arabidopsis transcriptome containing over 82k unique transcripts and thereby far greater diversity of alternatively spliced isoforms than TAIR10 and Araport11 (Zhang et al., 2017). LncRNA gene lists from Liu et al. (2012), PLncDB:Plant Long non-coding RNA Database, lncRNAs from TAIR, and Araport and antisense lncRNAs from Araport were compared to AtRTD2 giving 379 lncRNA genes.

### Quantitative Reverse Transcription RT-PCR (RT-qPCR)

Real-time RT-PCR was performed essentially as described previously (James et al., 2012). Complementary DNA (cDNA) was synthesized from 2 μg of total RNA using oligo dT primers and SuperScriptII reverse transcriptase (ThermoFisher Scientific). Each reaction (1:100 dilution of cDNA) was performed with Brilliant III SYBR Green QPCR Master Mix (Agilent) on a StepOnePlus (Fisher Scientific-UK Ltd., Loughborough, United Kingdom) real-time PCR system. The average Ct values for *IPP2* (AT3G02780) were used as internal control expression levels. The delta–delta Ct algorithm (Livak and Schmittgen, 2001; James et al., 2012) was used to determine relative changes in gene expression. Primers TAS1a-ex1-fwd 5′-CTAAGCGGCTAAGCCTGACGTCA-3′ and TAS1a-ex2-ex1-rev 5′-CACCCATTACAAGCCTTTCTATCAGACAAGAC-3′ targeted spliced *TAS1a* transcripts where the latter primer bridged the spliced intron (between exonic nucleotides underlined in the primer sequence). Primers amplifying total *TAS1a* transcripts comprised the aforementioned TAS1a-ex1-fwd primer in combination with primer TAS1a-ex1-rev 5′-CAGACAAGACCATGACTCGATCTAAAGGC-3′.

### High-Resolution (HR) RT-PCR

High-resolution (HR) RT-PCR reactions were conducted as described previously (Simpson et al., 2008). Gene-specific primer pairs (Supplementary Table S3) were used for analyzing the expression and AS of different genes. For each primer pair, the forward primer was labeled with 6-carboxyfluorescein (FAM). cDNA was synthesized from 4 μg of total RNA using the Sprint RT Complete – Double PrePrimed Kit following manufacturer’s instructions (Clontech Laboratories, Takara Bio Company, United States). The PCR reaction usually contained 3 μL of diluted cDNA (1:10) as a template, 0.1 μL of each of the forward and reverse primers (100 mM), 2 μL of 10 X PCR Buffer, 0.2 μL of Taq Polymerase (5 U/μL, Roche), 1 μL of 10 mM dNTPs (Invitrogen, Life Technologies), and RNase-free water (Qiagen) up to a final volume of 20 μL. For each reaction, an initial step at 94°C for 2 min was used followed by 24–26 cycles of (1) denaturation at 94°C for 15 s, (2) annealing at 50°C for 30 s, and (3) elongation at 70°C for either 1 min (for fragments smaller than 1000 bp) or 1.5 min (for fragments between 1000 and 1200 bp) and a final extension cycle of 10 min at 70°C. To separate the RT-PCR products, 1.5 μL of PCR product was mixed with 8.5 μL of Hi-Di^TM^ formamide (Applied Biosystems) and 0.01 μL of GeneScan^TM^ 500 LIZ^TM^ dye or 0.04 μL of GeneScanTM 1200 LIZ^TM^ dye size standard and run on a 48-capillary ABI 3730 DNA Analyser (Applied Biosystems, Life Technologies). PCR products were separated to single base-pair resolution and the intensity of fluorescence was measured and used for quantification in relative fluorescent units (RFUs). The different PCR products and their peak levels of expression were calculated using the Genemapper^®^ software (Applied Biosystems, Life Technologies).

### Small RNA Extraction and Detection of miRNAs and siRNAs

Total RNA was extracted from rosette material using TRI Reagent reagents (Sigma–Aldrich, United States) according to the manufacturer’s instructions. For RNA gel blot hybridization of small RNAs 10 μg of total RNA was separated on a 15% polyacrylamide (19:1) gel with 8 M urea and 1 × MOPS (20 mM MOPS/NaOH, pH7) buffer. RNA markers (Decade RNA markers, Ambion, United States) were end-labeled by [^32^P]γ-ATP according to the manufacturer’s instructions. RNA was blotted onto Hybond-N membrane (Amersham, GE Healthcare, United Kingdom) using a Panther^TM^ Semi-dry Electroblotter, HEP-1 (Thermo Scientific Owl Separation Systems, United States) and cross-linked by *N*-(3-dimethylaminopropyl)-*N*′-ethylcarbodiimide hydrochloride (EDC, Sigma–Aldrich, United States; Pall et al., 2007). DNA oligos (20 pmol) were end-labeled by [^32^P]γ-ATP using T4 polynucleotide kinase (NEB, United States) to visualize small RNAs. Hybridization was performed in PerfectHyb^TM^ Plus Hybridization Buffer (Sigma–Aldrich, United States). After overnight incubation at 37°C, the membrane was washed twice in 2 X SSC and 0.1% SDS for 15 min at 37°C. After washing, signals were detected by phosphorimager plate visualized by FLA-7000 Fluorescent Image Analyzing System (Fujifilm, United States). The scanned images were quantified by AIDA Image Analyzer software (Fujifilm, United States). The same membrane was re-hybridized with the different probes; it was stripped after each hybridization using 0.1 × SSC, 0.1% SDS at 65°C for 30 min and the efficiency was checked by overnight exposure. Student’s *t*-test (*p* < 0.05) was used to identify differentially expressed small RNAs.

## Results

### Identification of Cold-Induced Changes in Expression and Alternative Splicing

To examine changes in gene expression and AS in response to low temperature, we previously performed deep RNA-seq on a diel time-series of 5-week-old Arabidopsis Col-0 rosettes grown at 20°C and transferred to 4°C (Supplementary Figure S1A; Calixto et al., 2018). Briefly, rosettes were sampled at 3 h intervals for the last day at 20°C, the first day at 4°C, and the fourth day at 4°C (Supplementary Figure S1A) and each time-point consisted of three biological replicates. Over 360 million paired-end reads were generated for each of the 26 time-points and transcript abundances were quantified using Salmon (Patro et al., 2017) and AtRTD2-QUASI as the reference transcriptome (Zhang et al., 2017). The time-series data was analyzed at both the gene and individual transcript levels to identify genes with significant DE and significant differential alternative splicing (DAS). Briefly, this was achieved by generating read counts data using tximport (Soneson et al., 2015), normalizing data across samples with edgeR (Robinson et al., 2010), transforming using the voom function in limma (Law et al., 2014; Ritchie et al., 2015; Law et al., 2016), and establishing contrast groups in limma (for details see section “Materials and Methods” and Calixto et al., 2018). The experimental design allowed direct comparisons between equivalent time-points at 20°C and those in day 1 or day 4 at 4°C. This controlled for any effects of time-of-day variation in expression so that the changes detected were due to reduction in temperature. The statistical criteria for a DE gene were that it must have a log2-fold change ≥ 1 (≥2-fold change) in expression in at least two consecutive contrast groups with an adjusted *p*-value of <0.01. To detect DAS genes, the consistency of expression changes between the total expression of the gene and individual transcripts was examined using *F*-tests. For a gene to be significantly differentially alternatively spliced, a log2-fold change value of at least one of the transcripts must differ significantly from the gene log2-fold change value with an adjusted *p*-value of <0.01, and show a Δ percent spliced (ΔPS) of ≥0.1 in at least two consecutive contrast groups (for details, see Calixto et al., 2018). Using these stringent criteria, we identified a total of 7302 DE genes and 2442 DAS genes whose expression was significantly differentially expressed or alternatively spliced, respectively. The overlap between DE and DAS genes was 795 genes being both significantly DE and DAS (regulated by both transcription and AS) in response to low temperature (Supplementary Figure S1B; Calixto et al., 2018).

### Cold-Induced Expression and AS of lncRNAs

The RNA-seq time-series data was analyzed using AtRTD2, a new transcriptome dataset for Arabidopsis (Zhang et al., 2017). DE and DAS analyses were therefore applied to the 379 lncRNA genes in AtRTD2. In general, for the majority of these lncRNA genes, AtRTD2 contained novel alternatively spliced transcripts or extended some of the shorter/truncated transcript models currently in the TAIR10/Araport11 thereby providing increased diversity of AS isoforms of the lncRNA genes.

**Figure 1:**
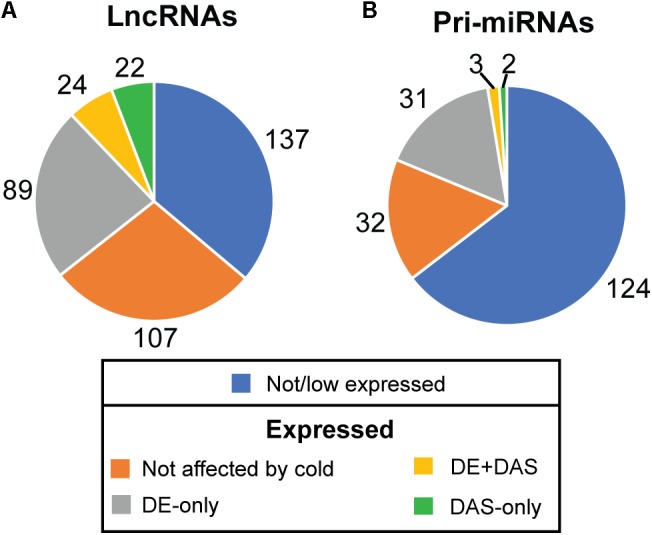
Expression and alternative splicing of lncRNAs and pri-miRNAs in response to cold. The number of **(A)** lncRNA and **(B)** pri-miRNA genes in RTD2 (Zhang et al., 2017) according to their expression in the RNA-seq time course and identifying the number which are differentially regulated by cold at the expression (DE) and/or alternative splicing level (DAS).

**Figure 2:**
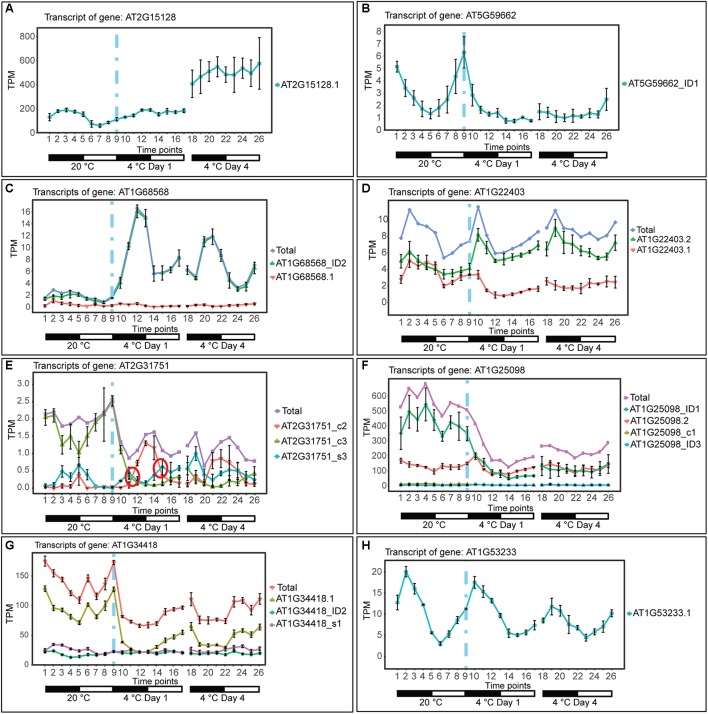
Expression of lncRNA transcripts in response to cold. Transcripts below 1.85 TPM in all time-points are not shown (except in **E**, where we used a 0.5 TPM cut-off value). Transition to cold is represented by a vertical dashed blue line at time-point 9. **(A)** The single transcript of AT2G15128 is DE showing a significant upregulation upon long exposure to cold. **(B)** AT5G59662_ID1 transcript is DE showing a significant downregulation upon cold and loss of a high amplitude rhythm is also observed. **(C)** The AT1G68568_ID2 transcript is DE showing a significant upregulation throughout the cold treatment and a gain of a high amplitude rhythm is also observed. **(D)** AT1G22403 (DAS-only) undergoes splicing regulation rapidly in the cold and the relative abundance of the AT1G22403.2 transcript is maintained in day 4 at 4°C (adaptive) whereas the total gene level is not significantly affected by cold. **(E)** AT2G31751 and **(F)** AT1G25098 are DE and DAS showing a significant downregulation upon cold. **(G)** AT1G34418 (DAS-only) undergoes only significant splicing regulation showing a rapid decrease in abundance of AT1G34418.1 and increasing expression during the day. **(H)** Rhythmic expression of AT1G53233 (not affected by cold). Rapid and significant isoform switches detected by TSIS (Guo et al., 2017) are labeled with a red circle. NAT lncRNA genes **(B,C,E,F,H)**; sORF **(G)**; other RNA **(A,D)**.

The depth of sequencing and the resolution of the RNA-seq time-course here allowed transcript-specific expression profiles of non-protein-coding genes to be analyzed. To examine the effect of low temperature on the expression and AS of lncRNAs, we searched the DE, DE+DAS, and DAS gene lists for the 379 lncRNA genes (see section “Materials and Methods”). Nearly a third of these genes (135) exhibited significant DE and/or AS in response to cold with 89 DE-only, 24 DE+DAS, and 22 DAS-only lncRNAs (Figure 1A and Supplementary Table S1A). Of these, 82 were NATs, eight encoded small open reading frames (sORFs; Hsu et al., 2016), two coded for tasiRNA precursors, and a third tasiRNA, *TAS4*, also encoded sORF27 (Supplementary Table S1B). The gene descriptions of the DE only, DE+DAS, and DAS only lncRNAs and of the genes overlapping the NAT lncRNAs are given in Supplementary Tables S1C–H. The expression profiles of DE lncRNAs showed a range of behaviors including cold-induced increase or decrease of expression, transient changes mainly seen in day 1 at 4°C, adaptive changes where the change in expression after transfer to cold persisted throughout the cold treatment, and late expression where changes were mainly observed in day 4 at 4°C (Figure 2). For example, AT2G15128 (DE) showed an increase in expression in day 4 at 4°C (Figure 2A) while AT5G59662 (DE) showed rhythmic expression at 20°C and expression decreased dramatically within the first 6–12 h after lowering the temperature (Figure 2B). AT2G15128 had one of the highest levels of expression increasing from around 200 TPM at 20°C to 450 TPM in day 4 at 4°C (Figure 2A). The DE gene, AT1G68568, had two transcripts which were expressed at relatively low levels at 20°C but the AT1G68568_ID2 transcript increased immediately upon onset of cold and showed rhythmic expression with the maximal level of expression phased to around the middle of the dark period (Figure 2C). For DAS genes (which had no significant DE at the gene level), significant changes in AS again showed a variety of expression profiles at the level of transcripts. For example, AT1G22403 had two transcripts which were expressed at similar levels at 20°C but the AT1G22403.2 transcript increased with cold with a concomitant decrease in the levels of AT1G22403.1 (Figure 2D). AT2G31751 is a DE+DAS gene with three main transcripts of which AT2G31751_c3 is the most abundant at 20°C peaking around dusk. At 4°C, its expression is drastically reduced and replaced by AT2G31750_c2 which becomes the most abundant transcript and has an altered expression pattern, peaking at dawn (Figure 2E). Two other lncRNAs (AT1G25098 – DE+DAS and AT1G34418 – DAS-only – encoding the sORF15 lncRNA) had two and three main transcripts, respectively (Figure 2F,G). In both cases, the most abundant transcript at 20°C decreased rapidly after the start of the cold treatment. Some lncRNA expression profiles were clearly rhythmic, peaking at different times of the day. For example, although not differentially expressed or differentially alternatively spliced, AT1G53233 had clear rhythmic profile across cooling with maximal expression shortly after the onset of dark (Figure 2H). Interestingly, in some cases, rhythmic expression was either dampened or amplified in the cold (Figure 2B,C, respectively).

To investigate possible functions of the NAT lncRNAs, a GO enrichment analysis of the gene descriptions of the potential protein-coding targets of the 84 DE, DE+DAS, and DAS NATs was performed. The only enriched terms were biological process: flavonoid glucuronidation and molecular function: UDP-glycosyltransferase activity, quercetin 7-*O*-glucosyltransferase and quercetin 3-*O*-glucosyltransferase activity (FDR cut-off < 0.05). The time-course also allowed the identification of genes which showed the largest and quickest changes in expression or AS (Calixto et al., 2018). Thirteen of the NAT targets were in this group and included three UDP-glycosyltransferase and a flavone-3-hydroxylase (consistent with the GO analysis) and three transcription factors: AT1G69570 – *CDF5*, AT5G18240 – *MYB-RELATED PROTEIN 1* (*MYR1*), and AT5G15850 – *CONSTANS-LIKE 1* (*COL1*). We also identified AS in three of the sORF-encoding lncRNA transcripts (AT3G344184 – *sORF15*; AT3G26612 – *sORF28*, and AT5G24735 – *sORF31*). Translation of the AS isoforms of all three showed that AS did not affect the presence or integrity of the sORF (not shown).

### Cold-Induced Expression and AS of pri-miRNAs

**Figure 3:**
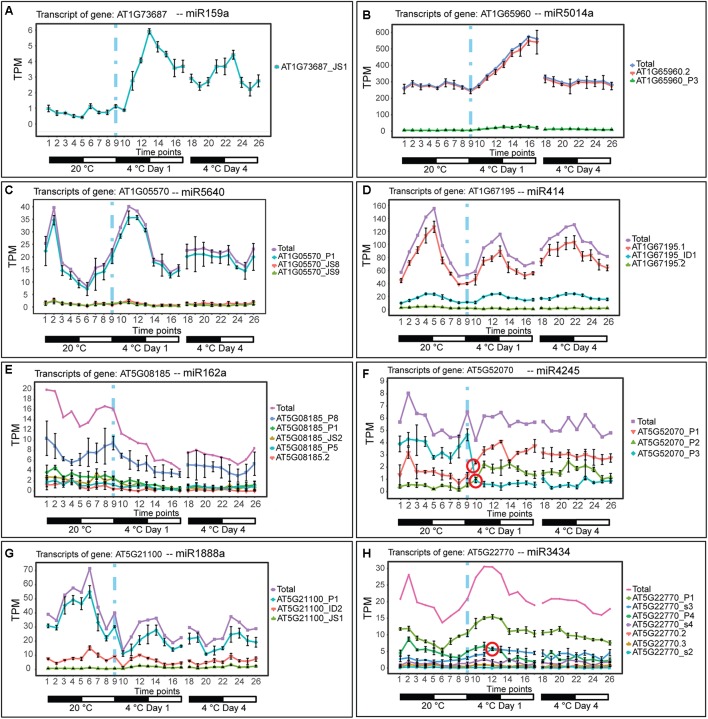
Expression of pri-miRNA transcripts in response to cold. Transcripts below 1.85 TPM in all time-points are not shown (except in **H**, where we used a 1 TPM cut-off value). Transition to cold is represented by a vertical dashed blue line at time-point 9. **(A)** pri-miR159a is DE showing a significant upregulation upon cold and a gain of a high amplitude rhythm is also observed. **(B)** pri-miR5014a, is an intronic miRNA in the *GLUTAMATE DECARBOXYLASE 2* gene (AT1G65960); the profile of the pre-mRNA is DE showing a significant upregulation only in the first day of cold treatment. **(C)** pri-miR5640 is an intronic miRNA in *CALLOSE SYNTHASE 1* (AT1G05570) which is DE in the cold, while upon longer exposure to cold (day 4) rhythmicity is lost/dampened. **(D)** pri-miR414 is rhythmically expressed and not significantly affected by cold. **(E)** pri-miR162a is DE and DAS showing a significant downregulation throughout the cold treatment. **(F)** pri-miR4245 is intronic in an *AGENET DOMAIN-CONTAINING PROTEIN* gene (AT5G52070) which undergoes splicing regulation with no significant expression change at the gene level. **(G)** pri-miR1888a is an intronic miRNA in an *L-ASCORBATE OXIDASE* gene (AT5G21100) that is downregulated at the transcriptional level and undergoes differential alternative splicing. **(H)** pri-miR3434 is an intronic miRNA in an *ALPHA-ADAPTIN* gene (AT5G22770) which undergoes only splicing regulation. Rapid and significant isoform switches detected by TSIS (Guo et al., 2017) are labeled with a red circle.

To investigate the effect of low temperature on the expression of pri-miRNAs, we identified the miRNA host genes that were DE-only, DE+DAS, and DAS-only. Using the miREx miRNA gene list (Bielewicz et al., 2012; Zielezinski et al., 2015), 192 pri-miRNA genes were present in AtRTD2 (Supplementary Table S2A). For the majority of these genes, AtRTD2 again contained novel alternatively spliced transcripts increasing the number of AS isoforms or extended some of the shorter/truncated transcript models currently in the TAIR10/Araport11. We detected expression of 68/192 pri-miRNA genes (Supplementary Table S2B). Of these, 31 were DE-only, three were DE+DAS, and two genes were DAS-only (Figure 1B). As with the lncRNAs, reducing the temperature caused increased or decreased expression at different time-points of the cold treatment. For example, the DE pri-miRNA gene, AT1G73687 (encoding miR159a) showed a concomitant rapid increase in expression and a diurnal waveform with a peak toward the end of the dark period in day 1 and day 4 at 4°C (Figure 3A). In contrast, the highly expressed pri-miRNA AT1G65960 (miR5014a) showed a transient increase in expression; levels doubled during Day 1 at 4°C but returned to the 20°C levels by day 4 at 4°C (Figure 3B). The main transcript of the DE pri-miRNA gene AT1G05570 (miR5640) showed rhythmic expression peaking in the night but appeared to lose rhythmicity by day 4 at 4°C (Figure 3C). Although not significantly affected by cold, the transcripts of pri-miRNA AT1G67195 (miR414) illustrate rhythmic expression (Figure 3D). Two DE+DAS pri-miRNA genes, AT5G08185 (miR162a) and AT5G21100 (miR1888a), showed reduction in the expression levels of their main transcripts with cold (Figure 3E,G). The DAS-only pri-miRNA gene, AT5G52070, encodes miR4245 (Figure 3F). At 20°C, AT5G52070_P3 is the most abundant transcript but shows a rapid decrease after onset of cold with a concomitant increase in the other two transcripts, AT5G52070_P1 and _P2 with isoform switches in the first 3 h of cold (Figure 3F). Finally, the DAS-only pri-miRNA gene, AT5G22770 (miR3434), had three main transcripts; AT5G22770_P1 and AT5G22770_s3 increased their expression slightly with cold while AT5G22770_P4 was decreased (Figure 3H). Of the 36 pri-miRNA genes with DE and/or differential AS, 13 were protein-coding genes containing an intronic miRNA (Supplementary Table S2B).

### Cold-Induced Alternative Splicing of *TAS1a*

**Figure 4:**
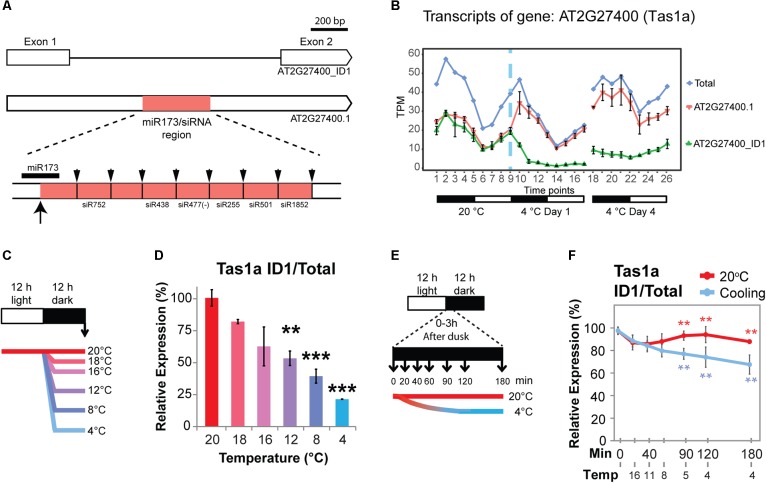
Cold-induced alternative splicing of Trans-acting siRNA 1a. **(A)** Transcript structures of *TAS1a* isoforms showing the intron and the position in the intron of the region containing the miR173 binding site and phased cleaved siRNAs. The up arrow shows the cleavage site of the *TAS1a* RNA by miR173 and the down arrows the cleavage sites which release the various siRNAs (Allen et al., 2005). **(B)** Expression profile of *TAS1a* transcript isoforms and gene; *TAS1a* is DAS-only (no significant change in gene level expression). **(C)** 5-week-old Arabidopsis rosettes harvested at dawn (arrow) after 12 h of variable reductions in temperature. Reductions (Δ) of 2, 4, 8, 12, and 16°C in temperature applied at dusk provides evidence of temperature-sensitive, long-term changes in AS. **(D)** Unspliced (intron retention – IR) of *TAS1a* (AT2G27400_ID1) is sensitive to reductions in temperature of 8°C. Student’s *t*-tests were performed to compare each temperature reduction results against 20°C control. **(E)** 5-week-old Arabidopsis rosettes harvested rapidly after transfer to cold. The temperature was gradually reduced from 20°C at 0 h to 11°C at 40 min and eventually 4°C at 120 min into the cold treatment; rosettes were harvested across the first 3 h of cold at the times shown allowing the measurement of the speed of transcriptional and AS changes due to temperature reduction. **(F)** The unspliced (IR) transcript of *TAS1a* (AT2G27400_ID1) responded rapidly to cold within 90 min, when the temperature reaches 5°C. RT-qPCR was used to measure relative expression levels for data presented in **D** and **F**, see Section “Materials and Methods.” Student’s *t*-tests were performed to compare each temperature reduction results against 20°C control. Significant differences are labeled with asterisks (^∗∗^*p* < 0.01; ^∗∗∗^*p* < 0.001).

*TAS1a* is an lncRNA that is regulated only by AS in response to cold (no significant DE at the gene level; Figure 4B). *TAS1a* RNA is initially targeted by miR173, converted to double-stranded RNA and subsequently cleaved into 21 nt phased small-interfering RNAs (phasiRNAs; Allen et al., 2005; Allen and Howell, 2010; Figure 4A). *TAS1a* and *TAS2* have both been reported to contain an intron (Vazquez et al., 2004; Yoshikawa et al., 2005). In AtRTD2, we confirmed the presence of the introns in *TAS1a* and *TAS2* and identified an intron in *TAS1c* which all contained the miRNA binding site and phasiRNAs (Supplementary Figures S2–S5). *TAS1a* produces two transcript variants: an unspliced transcript (AT2G27400.1) and a transcript where an intron is removed (AT2G27400_ID1). The intron contains the entire region of *TAS1a* which has the miR173 binding site and tasiRNAs (Figure 4A). Splicing of the intron was confirmed by RT-PCR using primers in exons 1 and 2 (Supplementary Figure S2A). In RNA-seq data, both transcripts were expressed at similar levels at 20°C with higher levels of expression during the night (Figure 4B). Upon temperature reduction, there was a rapid decrease of the spliced isoform (AT2G27400_ID1, Figure 4B) in the first 6 h after the start of cold application, while the unspliced AT2G27400.1 increased in the first 3 h and then showed a decrease over the next 12 h. In day 4 at 4°C, the divergent levels of the unspliced and spliced isoforms relative to the 20°C patterns were maintained. The change in splicing ratio (ΔPS – percent spliced) was > 0.3 such that *TAS1a* is one of 137 DAS genes with the quickest and largest responses to cold (Calixto et al., 2018). To investigate the sensitivity of AS of *TAS1a* to temperature reductions, plants were exposed to incremental reductions in temperature involving step-wise drops of between 2 and 16°C from the starting temperature of 20°C (Figure 4C). Isoform abundances were measured using isoform-specific RT-qPCR (Figure 4C,D). We observed that the spliced isoform (AT2G27400_ID1) decreased rapidly with lower temperatures showing a significant change with only an 8°C reduction in temperature (20–12°C; Figure 4D). To examine the speed and sensitivity of AS of *TAS1a*, we used RT-qPCR to measure changes in AS in plants after 20, 40, 60, 90, 120, and 180 min of cold treatment (Figure 4E). Over this series of cooling time-points, we recorded the air temperature within the growth chamber such that after 40 and 60 min plants had experienced 11 and 8°C, respectively, and after 2 h, the temperature reached 4°C (Figure 4E,F). Similar to the previous experiment (Figure 4B), the spliced isoform (AT2G27400_ID1) was sensitive to the gradual reduction in temperature from 20 to 4°C and showed a significant reduction within the first 90 min into the cold when the temperature had reached approximately 5°C (Figure 4F). This data indicates that rapid and sensitive temperature-dependent AS plays an important role in regulating the expression behavior of *TAS1a* as seen previously for some protein-coding genes (Calixto et al., 2018).

**Figure 5:**
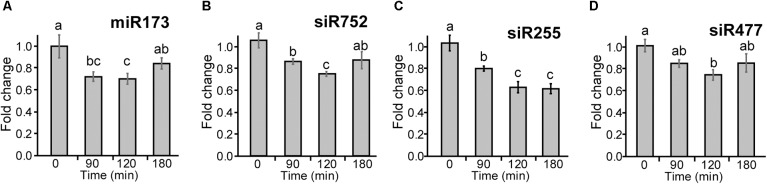
Cold-induced reduction of miR173 and *TAS1a*-derived siRNAs. Small RNA gel blot analysis was performed using RNA from rosette leaves harvested at different time-points after application of cold. Samples were hybridized with radioactively labeled probes specific to miR173 and the different siRNAs and bands were quantified. **(A)** miR173, **(B)** siR752, **(C)** siR255, and **(D)** siR477. The temperature at the different time-points was 20°C (0 min), 5°C (90 min), and 4°C (120 and 180 min). The letters on the bars indicate significantly different expression levels of small RNAs using Student’s *t*-test (*p* < 0.05); different letters mean significant differences between the samples.

To investigate whether the changes in abundance of *TAS1a* transcripts by AS affects the levels of mature siRNAs, we isolated small RNAs from 5-week-old Arabidopsis rosettes grown at 20°C in 12 h:12 h dark:light and decreased the temperature at dusk (Supplementary Figure S1A). Plants were sampled (three biological replicates) at 0, 90, 120, and 180 min after the temperature was reduced to 4°C. RNAs were isolated and separated on denaturing polyacrylamide gels, transferred to membrane, and hybridized with a series of different [^32^P]-labeled oligonucleotides specific to miR173 and siRNAs: siRNA752, siRNA255, and siRNA477 (Figure 5A–D, respectively, and Supplementary Figure S6) and U6snRNA as control. The intensity of hybridization signals was quantified using AIDA Image Analyzer software using U6snRNA as a loading control. Over the short cold exposure treatments, there was a significant decrease in the abundance of the miR173 and the siRNAs over the first 2 h (Figure 5). Thus, that decreasing temperature causes a reduction in the abundance of siRNAs derived from *TAS1a*.

### Dynamic Expression and AS of lncRNAs and pri-miRNAs

**Figure 6:**
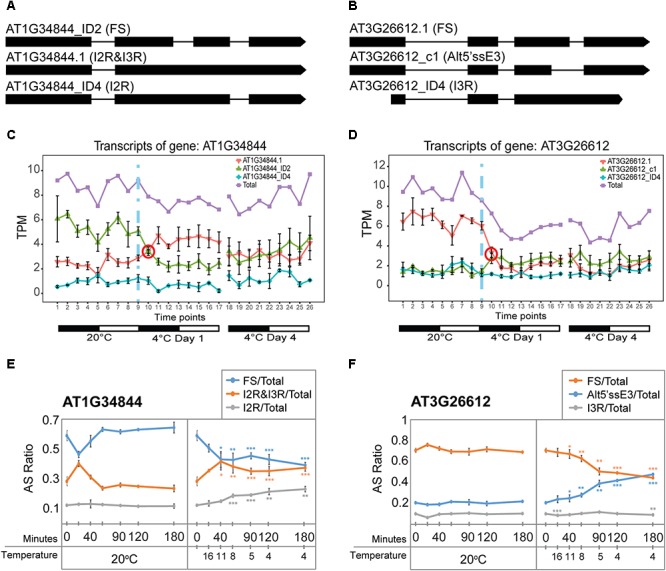
Rapid cold-induced alternative splicing of lncRNAs. **(A)** Transcript structures of AT1G34844 isoforms showing the fully spliced transcript (AT1G34844_ID2), AT1G34844.1 which retains introns 2 and 3, and AT1G34844_ID4 which retains intron 2. **(B)** Transcript structures of AT3G26612 isoforms showing the fully spliced transcript (AT3G26612.1), AT3G26612_c1 which has an alternative 5′ splice site in exon 3, and AT3G26612_ID4 which retains intron 3. **(C)** AT1G34844 is regulated by alternative splicing only. AT1G34844_ID2 and AT1G34844.1 show a transient decrease and increase, respectively, between 20°C and day 1 at 4°C while AT1G34844_ID4 remains unchanged throughout the experiment. **(D)** AT3G26612 is regulated by alternative splicing only. AT3G26612.1 significantly decreases in the first 6 h (day 1) and throughout the cold treatment, while the other two transcripts have similar levels of expression, with AT3G26612_c1 slightly increasing and AT3G26612_ID4 remaining unchanged. **(E,F)** High-resolution RT-PCR analysis of splicing ratios at 20°C (control – left panels) and decreasing to 4°C (right panels). **(E)** The fully spliced and I2R&I3R transcripts of AT1G34844 respond rapidly to changes in temperature within 40 min when the temperature reaches 11°C and onward as the temperature decreases further. **(F)** The fully spliced and Alt5′ssE3 transcripts of AT3G26612 respond rapidly to changes in temperature within 40 min when the temperature reaches 11°C and onward as the temperature decreases further, while I3R is mostly unresponsive. Student’s *t*-tests were performed to compare each temperature reduction results against 20°C control. Significant differences are labeled with asterisks (^∗∗^*p* < 0.01; ^∗∗∗^*p* < 0.001).

Besides *TAS1a*, other lncRNAs and pri-miRNA transcripts also showed rapid changes in expression and AS. As for *TAS1a*, the speed and sensitivity of such changes was also investigated using RNA from leaf samples collected during the first 3 h of cold treatment (see Figure 4E). The RNA-seq expression profile of the two main transcript isoforms of the NAT lncRNA, AT1G34844 (Figure 6A), showed that AT1G34844_ID2 decreased and AT1G34844.1 increased rapidly in the cold with an isoform switch in the first 3 h of cold (Figure 6C). In the expanded time-course covering the first 3 h when the temperature decreased gradually to 4°C, the AT1G34844.1 transcript (introns 2 and 3 retained) and AT1G34844_ID2 (fully spliced, protein-coding) showed significant increase and decrease, respectively, after only 40 min (11°C; Figure 6E, right panel) in comparison to the constant 20°C control (Figure 6E, left panel). AT3G26612 lncRNA (encoding sORF28) had three AS isoforms (Figure 6B) where AT3G26612.1 and AT3G26612_c1 switched in response to cold (Figure 6D). In the 3 h time-course, the two main transcripts of the AT3G26612 lncRNA (encoding sORF28) showed little difference in the 20°C control (Figure 6E, left panel) but significant changes in their abundance with temperature reduction. AT3G26612.1 decreased and AT3G26612_c1 increased rapidly with the first significant differences being detected at 40 min when the temperature had reached only 11°C (Figure 6F).

## Discussion

Dynamic changes in expression at both the gene and transcript levels occur in response to lowering temperature in Arabidopsis (Calixto et al., 2018). AS makes a major contribution to changes in the transcriptome with over a quarter of genes whose expression changes significantly undergoing AS in the cold. The dynamic contribution of AS was illustrated by the rapid cold-induced wave of AS activity accompanying the transcriptional response and further AS changes throughout the period of cold exposure. The analysis of the RNA-seq cold response time-course at the transcript level identified hundreds of protein-coding genes which were only regulated by AS with no significant changes in expression at the gene level: the majority of these genes were novel cold-responsive genes (Calixto et al., 2018). Here, we have focussed on DE and differential AS of lncRNAs and pri-miRNAs. We identified 135 cold-responsive lncRNAs which were significantly differentially expressed and/or differentially alternatively spliced. Of these, a third involved changes in AS which have not been described previously. In particular, the transcript level expression analysis identified cold-responsive DAS-only lncRNAs which would not be detected by microarrays or gene level RNA-seq analyses. Different lncRNAs showed different responses to cold with, for example, transient or adaptive changes in expression/AS and the AS of some lncRNAs responded extremely rapidly and to small reductions in temperature showing that the AS of these genes is temperature-sensitive. Finally, we identified cold-induced AS of *TAS1a* and that the processing of siRNAs from the primary transcript may be splicing dependent. Therefore, cold-induced AS occurs in many lncRNAs/pri-miRNAs but there is little knowledge of the molecular mechanisms by which AS modulates levels of lncRNAs or miRNAs or their downstream biological functions.

Pre-mRNA processing such as splicing, AS, and alternative polyadenylation can affect gene expression levels by controlling mRNA export and stability, and the production of different functional variants (particularly at the protein level). The interactions between these typical pre-mRNA processing steps, environmental stress, and lncRNA production have been relatively well studied in Arabidopsis pri-miRNAs (Kruszka et al., 2012; Yan et al., 2012; Bielewicz et al., 2013; Szweykowska-Kulińska et al., 2013; Barciszewska-Pacak et al., 2016; Knop et al., 2017; Stepien et al., 2017). Plant miRNAs are mostly exonic and encoded by independent transcription units of which >50% contain introns (Szarzynska et al., 2009). Both splicing of introns downstream of the miRNA and the integrity of proximal 5′ splice sites have been shown to be required for efficient miRNA production (Bielewicz et al., 2013; Knop et al., 2017; Stepien et al., 2017). Mutations in various splicing factor genes including STA1 and the cold-induced GRP7 impact miRNA biogenesis from intron-containing pri-miRNAs (Ben Chaabane et al., 2013; Köster et al., 2014). GRP7, in particular, caused a reduction in the levels of many miRNAs along with accumulation of pri-miRNAs; direct binding of GRP7 was required to inhibit pri-miRNA processing. GRP7 also directly affected the splicing/AS of two pri-miRNAs (Köster et al., 2014). Furthermore, in Arabidopsis, 29 miRNAs are encoded within introns in protein-coding or non-coding host genes (Brown et al., 2008; Yan et al., 2012) and miRNA biogenesis is affected by splicing/AS and alternative polyadenylation via various mechanisms. For example, AS can remove regions of the pre-miRNA affecting its ability to fold correctly and be processed, and conversely, pre-miRNA secondary structure can affect splice site choice (Brown et al., 2008; Barciszewska-Pacak et al., 2016; Stepien et al., 2017). Production of miR400, an intron-located miRNA, was dependent on splice site choice: an AS event which removed part of the intron and left the miR400 in the mRNA of the host gene was induced by heat and led to an increase of the host gene mRNA containing the miRNA and a decrease in abundance of mature miR400 (Yan et al., 2012). The AS event effectively changed the position of the pre-miRNA from intronic to exonic (Yan et al., 2012; Szweykowska-Kulińska et al., 2013) suggesting that miRNA production was splicing-dependent and regulated by temperature. On the other hand, the intronic miRNA, miR402, is also induced by heat and correlates with selection of an intronic alternative polyadenylation site which competes with splicing and the miRNA is processed from the alternatively polyadenylated transcript (Knop et al., 2017). Thus, splicing and AS of pri-miRNAs impact the processing and levels of mature miRNAs and the AS of some pri-miRNAs is affected by cold.

Far less is known about AS in plant lncRNAs. Here, we exploited the increased number of transcript isoforms in AtRTD2 to identify novel AS events in Arabidopsis lncRNAs in the cold. In the analysis of the dynamics of AS in response to cold, we identified genes which showed the largest (ΔPS > 0.25) and quickest (0–6 h of cold) changes in cold-induced AS (Calixto et al., 2018). One of these genes encoded the tasiRNA lncRNA, *TAS1a*, which showed a rapid decrease in the level of the spliced isoform which correlated with a reduction in siRNAs. This suggested that splicing was required for the generation of the *TAS1a*-encoded siRNAs and was consistent with the earlier observation that a T-DNA insertion into the *TAS1a* intron led to greatly decreased siRNA production (Vazquez et al., 2004). Previously, reduced levels of *TAS1a*-derived siRNAs were observed in the cold while the levels of *TAS1a* were unaffected (Kume et al., 2010). Here, we also find reduced levels of the siRNAs in the cold but the transcript level analysis is able to distinguish the underlying AS. *TAS1c* and *TAS2* also have introns that contain the miRNA binding site and siRNAs but while the *TAS2* intron is efficiently spliced, *TAS1c* is spliced at very low frequency (Supplementary Figures S2–S5). The different organization of the TAS RNAs suggests that splicing may be important for efficient production of siRNAs for some genes but what determines why some genes undergo AS and the balance between splicing and processing are unknown.

The functions of the majority of Arabidopsis lncRNAs and, in particular, those with AS are still to be determined. The levels of *TAS1a*-derived siRNAs are affected in different environmental conditions and lower levels are detected in salt, drought, and cold stress (Sunkar and Zhu, 2004; Kume et al., 2010). The siRNAs derived from *TAS1a* have five gene targets of largely unknown function. However, the *TAS1a* siRNA target genes *AT1G51670*, *AT5G18040*, and *AT4G29760* are upregulated at 4°C with the increase in AT1G51670 due to reduced silencing by tasiRNAs (Kume et al., 2010). Two of the targets, *AT4G29770* and *AT5G18040* (*HEAT-INDUCED TAS1 TARGET1 and 2 – HTT1 and HTT2*), are upregulated in heat stress and mediate thermotolerance (Li et al., 2014). Plants over-expressing *TAS1a* produced higher levels of *TAS1a* siRNAs, reduced expression of *HTT1* and *HTT2*, and showed weaker thermotolerance than wild-type (Li et al., 2014). Here, we show cold-induced reduction in *TAS1a*-derived siRNAs and significant upregulation of *HTT2*. Thus, *TAS1a*-derived siRNAs may modulate expression of genes regulating the response of plants to both increased and decreased temperatures.

Other lncRNAs include natural antisense RNAs which generate dsRNA for silencing their target mRNAs, or lncRNAs which interact with protein splicing factors or chromatin modification factors to affect AS and expression of downstream genes (Romero-Barrios et al., 2018). We have identified many cold-responsive NAT lncRNAs with altered expression/AS and around half of the protein-coding genes that overlap these NATs were also differentially expressed and/or differentially alternatively spliced suggesting that the regulation of at least some protein-coding targets involve NAT lncRNAs. The GO enrichment analysis of the NAT targets of the DE/DAS lncRNAs identified genes involved in biosynthesis of flavonoids, secondary metabolites with a range of functions involved in growth, physiology, detoxification, and possibly acting as scavengers of reactive oxygen species (Pourcel et al., 2007). Some of these genes also had the earliest detectable DE which may reflect the upregulation of many genes involved in tolerance to reactive oxygen species early in response to chilling temperatures (Knight and Knight, 2012). In addition, two transcription factor NAT targets with the largest and most rapid changes in expression were involved in flowering control: *CDF5* and *MYR1*. The lncRNA, *FLORE*, is a natural antisense RNA which oscillates, has antiphasic expression to its *CDF5* mRNA target, and regulates flowering time (Henriques et al., 2017). Interestingly, *FLORE* is alternatively spliced into four isoform variants but the role of this AS is unknown. MYR1 is a G2-like protein containing an N-terminal MYB-like domain, a central coiled-coil domain, and a C-terminal transactivation domain. MYR1 is a negative regulator of flowering time under low light (Zhao and Beers, 2013). It has conserved alternative 3′ splice sites events which affect a highly conserved sequence in the coiled coil domain and homo-dimerisation properties or interactions with other transcription factors (Zhao and Beers, 2013). The *MYR1* isoforms increase rapidly over the first 6 h of cold treatment and then decrease 12–15 h after onset of cold. The *MYR1 NAT* (AT5G18245) peaks in the dark at 20°C and rapidly decreased in the first 3–6 h of cold. Thus, the *MYR1 NAT* and *MYR1* AS transcripts appear to be anti-phasic and the increase of isoforms may produce transcription factor complexes which act to suppress flowering. The regulation of *CDF5* and *MYR1*, both involved in flowering, by NATs, parallels the recent report of the NAT lncRNA, *MAS*, which is induced by cold and required for activation of *MAF4* expression and suppression of precocious flowering (Zhao et al., 2018). Therefore, one function of the changes in expression and/or AS of NAT lncRNAs in response to cold is suppression of flowering. The expression profiles of some Arabidopsis pre-mRNAs and lncRNAs showed altered patterns of rhythmic expression in response to cold (Calixto et al., 2018; Figure 2). A third NAT-associated transcription factor with the largest and fastest changes in expression, *COL1* is involved in regulation of specific circadian rhythms (Ledger et al., 2001). Over-expression of COL1 shortened the period of by 2–3 h. Here, both the *COL1 NAT* and *COL1* showed a large transient increase in expression in day 1 at 4°C with the peak of expression advanced by around 3 h compared to 20°C. Thus, the increase in expression and altered timing of the NAT correlates with the cold response of COL1 and may contribute to altered circadian control of expression of other genes in response to cold. Finally, although not included in this study, transcription of the lncRNA *SVALKA-asCBF1* which overlaps CBF1 suppresses CBF1 expression and impacts cold acclimation (Kindgren et al., 2018).

Plants experience constantly changing temperatures on hourly, daily, and seasonal scales and must have flexible regulatory systems to modulate gene expression. Gene regulation is complex involving the interplay between transcription and various post-transcriptional processes. AS is a major contributor to the changes in expression in the cold response (Calixto et al., 2018). In this paper, we demonstrate cold-induced changes in expression and AS of pri-miRNAs and lncRNAs and that AS of some lncRNA occurs very rapidly and are highly temperature sensitive. It is therefore likely that AS impacts the expression of target genes to contribute to both short term temperature responses and cold acclimation. A key question is the role of AS in regulating the processing, levels, and function of lncRNAs. The recent upsurge of interest in the role of intron retention in regulation of expression in plants and animals may provide possible explanations for splicing/AS of some lncRNAs. Intron retention can cause transcripts to be retained in the nucleus to wait for a splicing signal or be degraded while splicing can activate and enhance export of the transcripts to the cytoplasm where they can interact with target mRNAs to affect translational efficiency or be degraded (reviewed by Jacob and Smith, 2017). Thus, splicing/AS of lncRNAs may be a mechanism for regulating the levels of lncRNAs in the nucleus or cytoplasm or the stability of the lncRNAs which will impact the expression of target or downstream genes. Rapid changes in splicing factor activity, levels, or nuclear localization in response to cold can affect the efficiency of splicing/AS and determine whether different transcript isoforms are in the nucleus or cytoplasm affecting their processing or translation (e.g., sORF lncRNAs). Such mechanisms will require a thorough analysis of the transcript diversity of the thousands of plant lncRNAs to allow further studies on how the structure and processing pathways of different types of lncRNAs influence their localization, stability, and function.

## Data Availability

Publicly available datasets were analyzed in this study. This data can be found here: https://www.ebi.ac.uk/ena/data/search?query=prjeb19974.

## Author Contributions

JB, HN, and RZ obtained the funding support. JB and CC conceived and designed the experiments. CC, AJ, and NT collected and prepared samples. CC, AJ, NT, and CH performed the experiments. JB, CC, NT, AJ, and CH interpreted the main findings. CC and JB drafted the manuscript. All authors engaged in discussions during the project and revised and approved the final manuscript.

## Conflict of Interest Statement

The authors declare that the research was conducted in the absence of any commercial or financial relationships that could be construed as a potential conflict of interest.

## Footnotes

1 https://pypi.python.org/pypi/cutadapt/1.4.2
