# Supplementary material for: Cold-Dependent Expression and Alternative Splicing of Arabidopsis Long Non-coding RNAs
Source: Front Plant Sci. 2019 Feb 28;10:235. doi: 10.3389/fpls.2019.00235 (PMC6413719; doi:10.3389/fpls.2019.00235)
Supplement: Supplementary file 2 [file Data_Sheet_1.pdf]

Supplementary Figures

Cold-Dependent Expression and Alternative Splicing of Arabidopsis Long Non-Coding RNAs

Cristiane P. G. Calixto, Nikoleta A. Tzioutziou, Allan B. James, Csaba Hornyik, Wenbin Guo, Runxuan Zhang, Hugh G. Nimmo and John W. S. Brown*

*** Correspondence**: j.w.s.brown@dundee.ac.uk

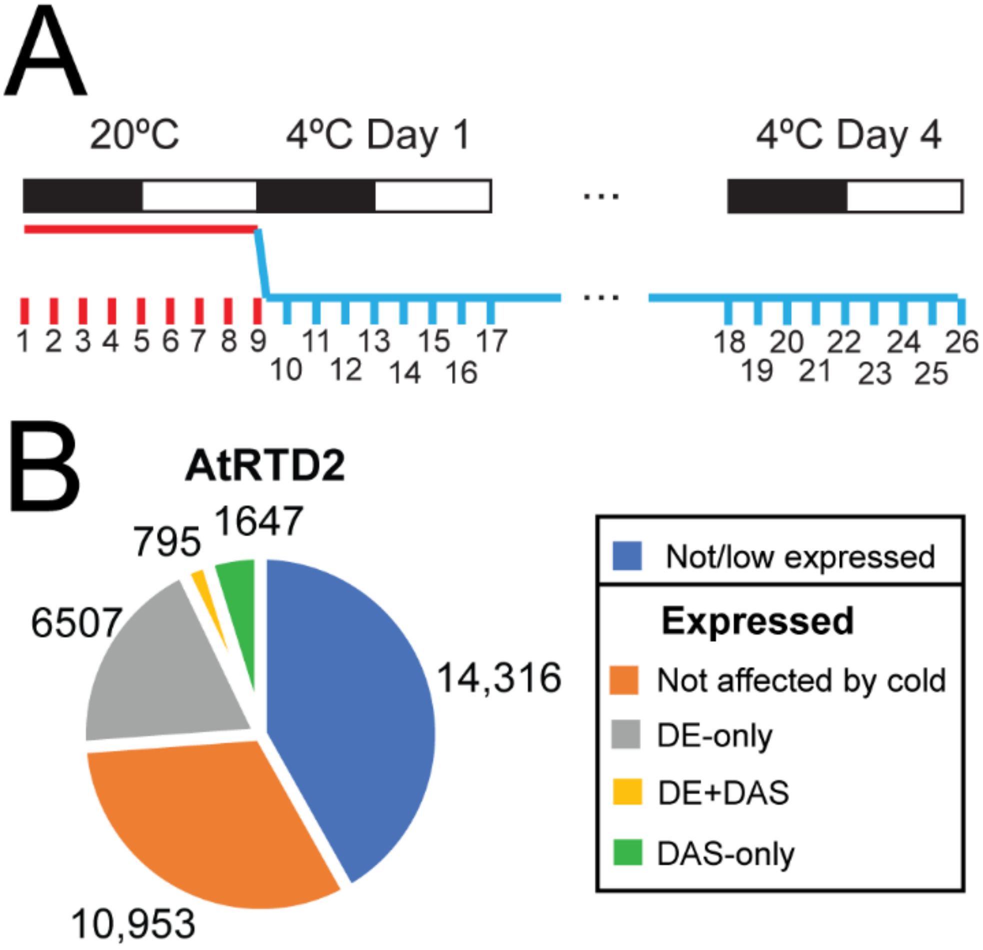

Supplementary Figure S1. RNA-seq time-course analysis of the Arabidopsis response to low temperature in diel conditions from Calixto et al. (2018). (A) Sampling strategy. Time-points of sampling are marked by vertical coloured lines and labelled from 1 to 26. Five-week-old Arabidopsis rosettes were harvested every 3 h over a 24 h-period at 20°C (red lines). At dusk, the temperature was gradually reduced to 4°C (blue lines), and harvesting continued during the first day at 4°C and the fourth day at 4°C. Black boxes, 12 h dark; white boxes, 12 h light. **(B)** Number of AtRTD2 genes not/low expressed, expressed but not significantly affected by the cold treatment, differentially expressed (DE) and/or differentially alternatively spliced (DAS) [results are redrawn from Calixto et al. (2018)].

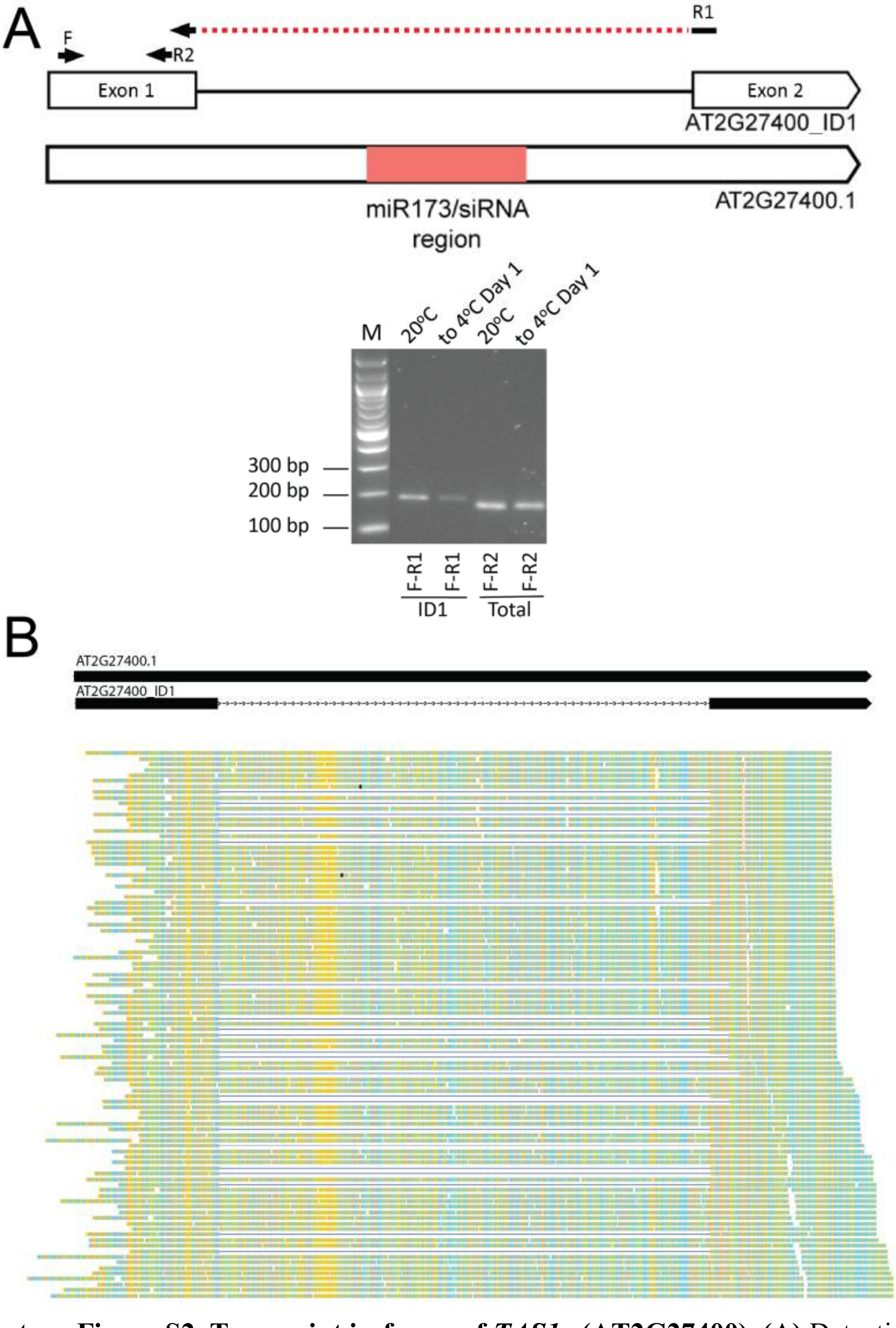

Supplementary Figure S2. Transcript isoforms of *TAS1a* (AT2G27400). (A) Detection of the spliced transcript isoform, AT2G27400_ID1 using RT-PCR. RNA from samples harvested at dawn on the last day at 20°C and on the first day at 4°C were used for the analysis. The same forward primer (F) was used in all reactions; R1 spanned the exon 1-exon 2 splice junction and was specific to the spliced isoform, AT2G27400_ID1, while R2 detected both the unspliced and spliced transcripts. PCR product sizes were predicted to be 168 and 146 bp for AT2G27400_ID1 and both transcripts, respectively. Amplicons were co-electrophoresed with 100 bp markers (M) (New England BioLabs) on a 1.5% (0.5x TBE) agarose gel. F = Forward primer, R = Reverse primer. **(B)** Alignment of RNA-seq reads covering the whole length of the gene using IGB (Nicol et al., 2009) with schematic diagrams of the two transcripts; reads supporting the splicing of the intron (blue lines).

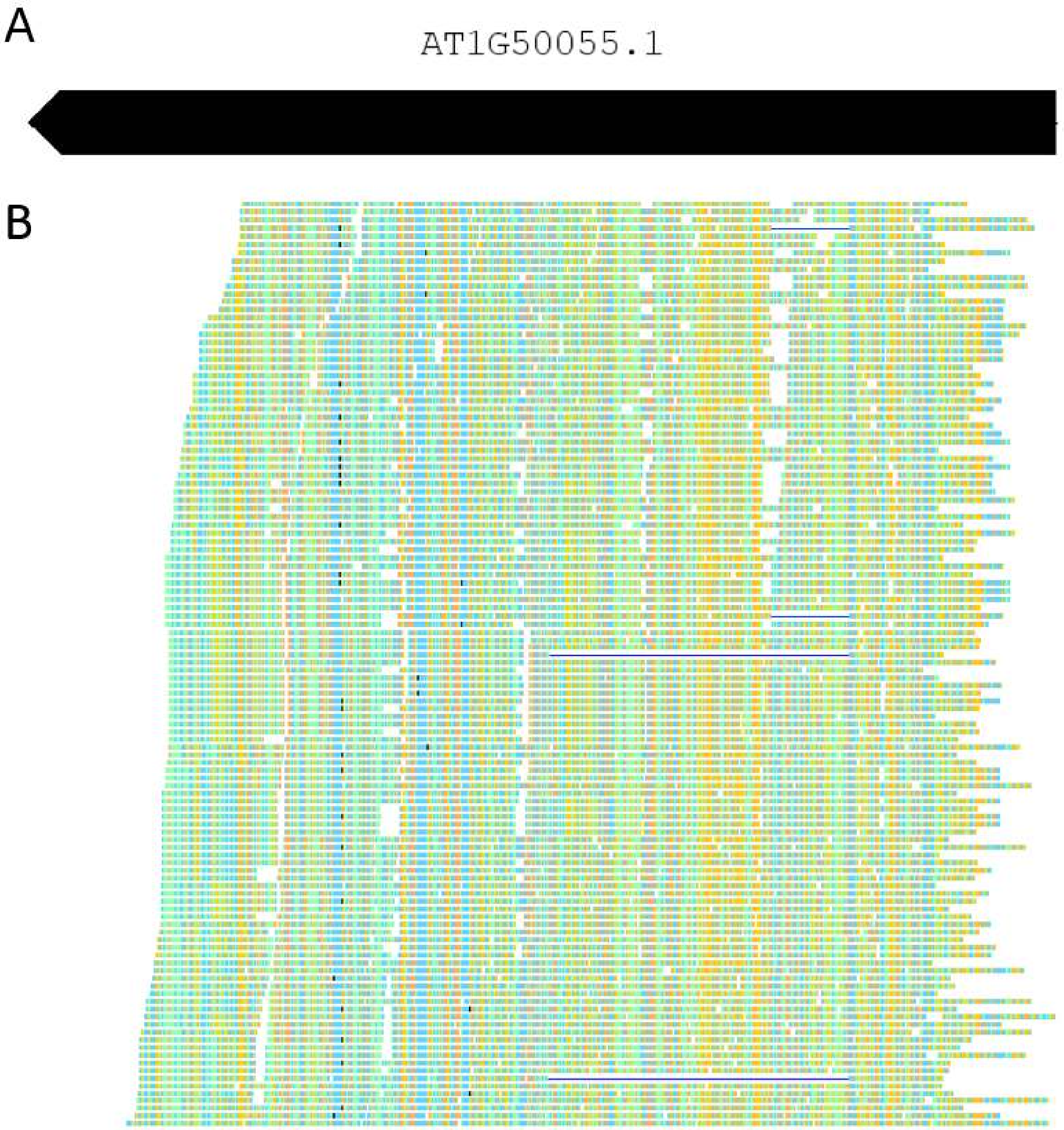

Supplementary Figure S3. Transcript of *TAS1b* (AT1G50055). (A) AT1G50055 is a single exon gene in AtRTD2. **(B)** Alignment of RNA-seq reads covering the whole length of the gene using IGB (Nicol et al., 2009).

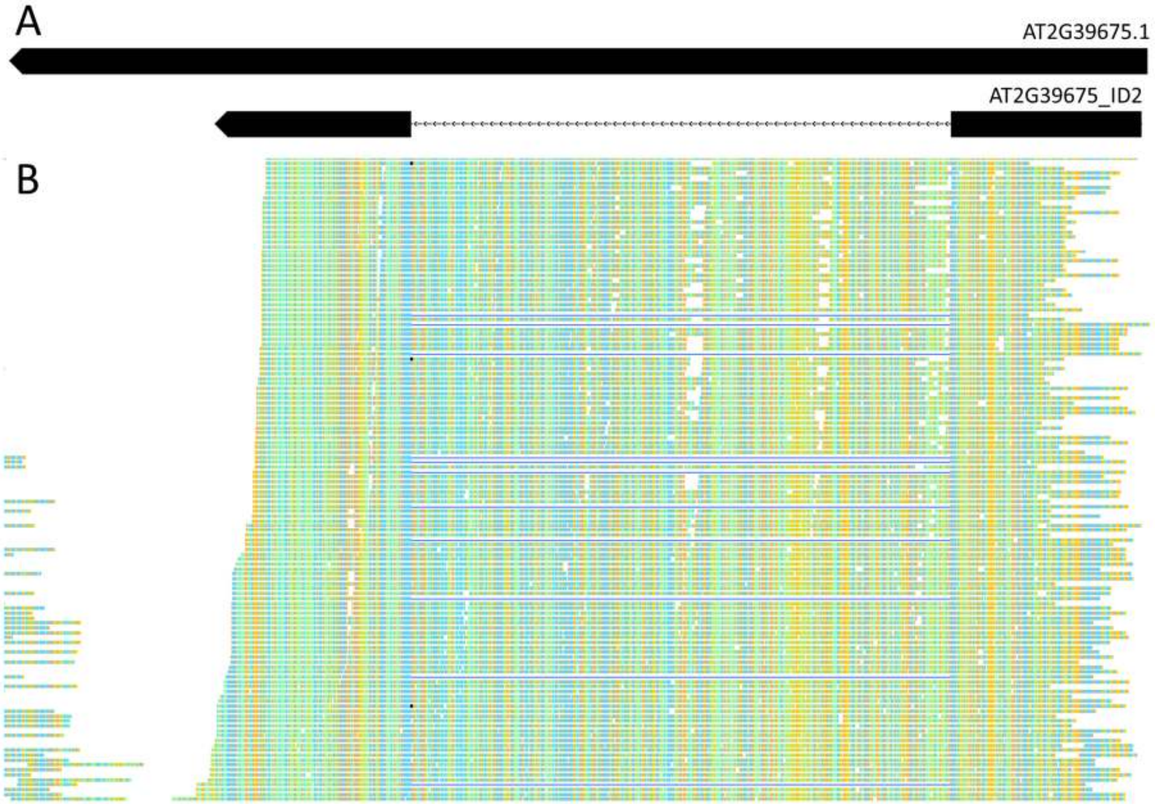

Supplementary Figure S4. Transcript isoforms of *TAS1c* (AT2G39675). (A) AT2G39675 has two different alternative isoforms: AT2G39675.1 which retains the intron and AT2G39675_ID2 which splices the intron out. **(B)** Alignment of RNA-seq reads covering the whole length of the gene using IGB (Nicol et al., 2009); reads supporting the splicing of the intron (blue lines).

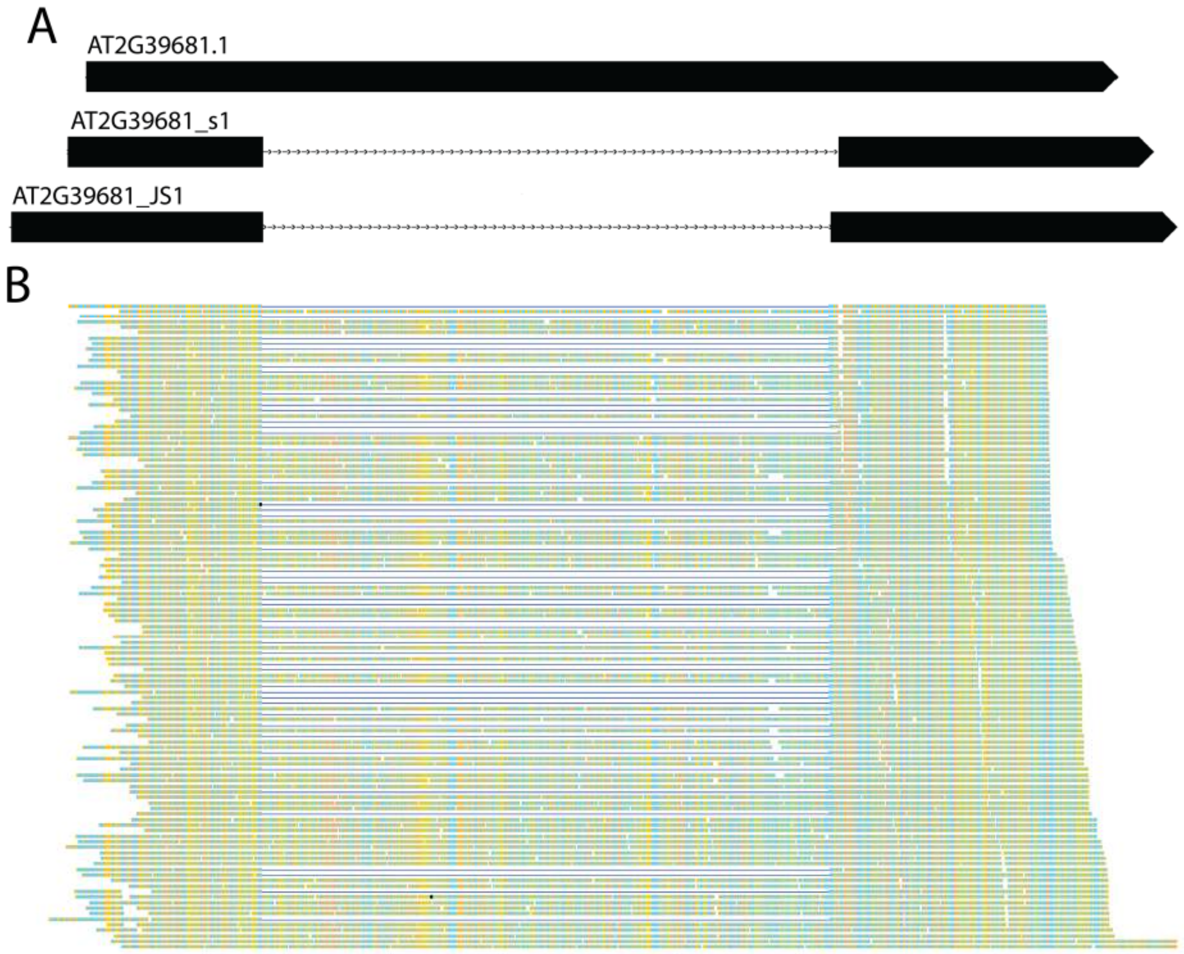

Supplementary Figure S5. Transcript isoforms of *TAS2* (AT2G39681). (A) AT2G39681 has three different alternative isoforms: AT2G39681.1 which retains the whole intron, AT2G39681_JS1 and AT2G39681_s1 which splice out the intron. AT2G39681_JS1 and AT2G39681_s1 differ by an alternative 3' splice site in Exon 2. **(B)** Alignment of RNA-seq reads covering the whole length of the gene using IGB (Nicol et al., 2009); reads supporting the splicing of the intron (blue lines).

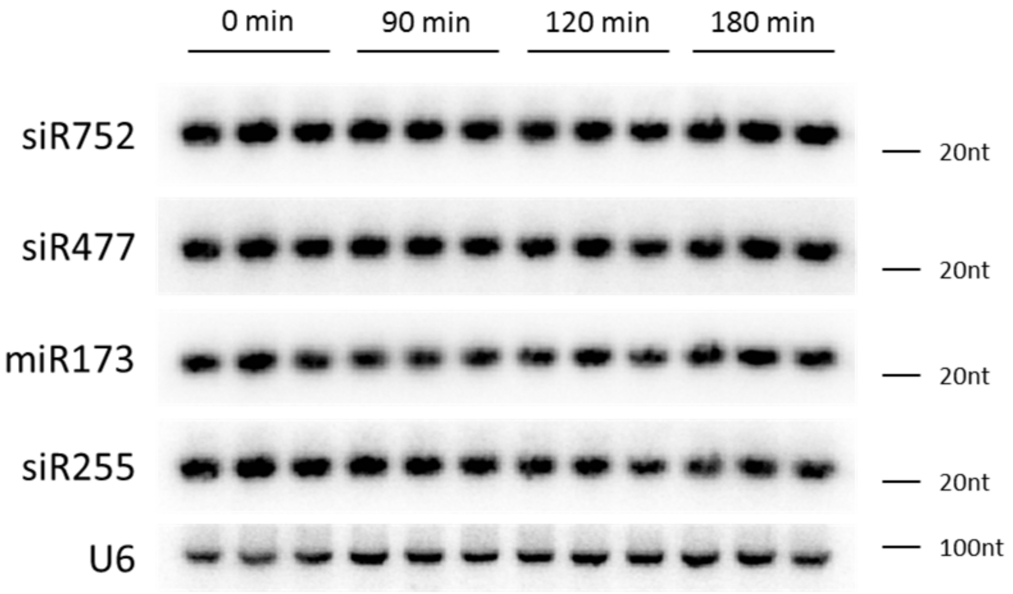

Supplementary Figure S6. Small RNA blot of expression of miR173 and phasiRNAs. Total RNA from three replicates of four time-points at and after decreasing temperature (see text for details) was hybridised with different probes to detect miR173, three siRNAs (siR752, siR477 and siR255) and the U6snRNA loading control.

Reference

Calixto, C.P.G., Guo, W., James, A.B., Tzioutziou, N.A., Entizne, J.C., Panter, P.E., Knight, H., Nimmo, H.G., Zhang, R., and Brown, J.W.S. (2018). Rapid and dynamic alternative splicing impacts the Arabidopsis cold response transcriptome. Plant Cell 30, 1424-1444.

Nicol, J.W., Helt, G.A., Blanchard, S.G.J., Raja, A., and Loraine, A.E. (2009). The Integrated Genome Browser: free software for distribution and exploration of genome-scale datasets. *Bioinformatics* 25**,** 2730-2731.
